# Perianal fistulas and the lift procedure: results, predictive factors for success, and long-term results with subsequent treatment

**DOI:** 10.1007/s10151-019-02023-9

**Published:** 2019-07-17

**Authors:** G. J. H. Vander Mijnsbrugge, R. J. F. Felt-Bersma, D. K. F. Ho, C. B. H. Molenaar

**Affiliations:** 1 Proctos Kliniek, Bilthoven, The Netherlands; 2 Department of Gastroenterology and Hepatology, Amsterdam UMC, Location VUmc, P.O. Box 7057, 1007 MB Amsterdam, The Netherlands

**Keywords:** Anorectal disease, Perianal fistula, Anal ultrasound, LIFT

## Abstract

**Background:**

Treatment of a perianal fistula is difficult due to the risk of fecal incontinence and recurrence. The ligation of intersphincteric tract (LIFT) procedure is a sphincter-saving procedure associated with success rates ranging from 57 to 94%. The aim of our study was to find predictors for a favorable outcome of the LIFT procedure, evaluation of postoperative fecal incontinence, quality of life, and subsequent treatment with long-term follow-up.

**Methods:**

This study was performed in patients who underwent LIFT between 2013 and 2015 at our institution. Their medical data were retrieved from the electronic patient files. The fistula characteristics were described by physical examination, three-dimensional endoanal ultrasound, and perioperative evaluation. Recurrence rate, postoperative fecal incontinence, and quality of life were assessed with the Patient-Reported Outcome Measurement (PROM). Thirty-two months later, long-term follow-up including subsequent procedures was evaluated.

**Results:**

Forty-five patients [17 men, mean age 40 years (range 24–67 years)] were included. In 41 (84%) patients, the fistula was classified as complex; 32 (71%) were referrals with a history of previous fistula surgery. The initial success rate was 18 (40%). Only the height of the internal fistula opening (≥ 15 mm *p* < 0.03) was associated with recurrence. The LIFT procedure did not affect the occurrence of fecal incontinence or soiling. Recurrence showed a trend with a lower PROM (*p* = 0.07). Twenty-four months later, further surgery leads to cure in 34 (75%), asymptomatic fistulas in 7 (16%), and persisting active fistulas in 4 (9%) patients.

**Conclusions:**

Initial LIFT had a success rate of 40% and with subsequent surgical treatment 75%. Recurrence after LIFT is related to the height of the internal fistula opening and is associated with diminished quality of life. Continence was not affected by initial LIFT.

## Introduction

A perianal fistula is an abnormal connection between the perianal skin and the anal canal or rectum [1]. It is probably an inflammatory condition in which infection begins in one of the 6–10 rudimental anal glands [2]. Recent insights suggest possible immunologic causes of a fistula [3].

Fistula treatment is complex due to possible recurrence and sphincter damage leading to soiling and fecal incontinence (FI). Laying open the tract by fistulotomy is still considered the most effective procedure. However, postoperative incontinence has been reported ranging from 4 to 62% and occurs generally around 13% [4]. For high and more complex fistulae (CF), approaches such as mucosal advancement flap (MAP) are recommended as the continence mechanism is more likely to become impaired after fistulotomy.

Primary fistulotomy and cutting setons are associated with the same incidence of GI depending on the complexity of the fistula, ranging from 25.2 to 67% [5, 6]. Although the aim of a surgical procedure is to cure a fistula, conservative management is sometimes warranted to preserve FI. However, trading radical surgery for sphincter-saving procedures such as a draining seton, fibrin sealant, anal fistula plug, and laser and Permacol^®^ instillation all result in more recurrence/persistence requiring repeated operations in many cases [7].

The ligation of intersphincteric fistula tract (LIFT) technique is the modified approach through the intersphincteric plane for the treatment of fistula-in-ano. The LIFT procedure is based on secure closure of the internal opening through the intersphincteric approach. Essential steps of the procedure include incision at the intersphincteric groove, identification of the intersphincteric tract, ligation of intersphincteric tract close to the internal opening, and removal of intersphincteric tract. Subsequently, the defect at the external sphincter muscle is sutured. The procedure was first described by Rojanasakul in 2007 [8, 9]. His preliminary results in terms of the success of this procedure were 94%. Later, several centers reported lower success rates. Although there are several studies about the recurrence rate after LIFT, data about incontinence and quality of life after LIFT are scarce. Furthermore, there is no consistency in the definition of the complexity of the treated fistulas and not much is known about the effect of certain specific characteristics such as the location of the internal fistula opening (IFO), the height of the IFO measured from the ano-dermal junction, and use of certain surgery techniques, e.g., ligation versus suturing of the fistula tract on the outcome.

The aim of this study was to determine if these specific characteristics are relevant to the outcome of recurrence, FI, and quality of life (QOL), and the long-term outcome after subsequent procedures.

## Materials and methods

### Patients and clinic

The Proctos clinic is specialized in proctologic surgery and serves as a referral clinic.

Inclusion criteria were all consecutive patients with perianal fistulas who were treated with an LIFT procedure within the period of January 2013 (when the clinic started using the procedure) to December 2015. Treatment was offered based on shared decision-making.

Exclusion criteria were patients with an intersphincteric fistula, an abscess, rectovaginal fistulas, fistula due to a pilonidal sinus, hidradenitis suppurativa, tuberculosis, human immunodeficiency virus infection, inflammatory bowel disease, actinomycosis, and anal carcinoma.

Data were gathered from the electronic patient file regarding demographics, symptoms, medical history, previous perianal surgical procedures, obstetric history, and findings of the proctological examination at first presentation.

### Three-dimensional endoanal ultrasound (3D-EAUS)

Physical examination was performed with the patient in the left lateral decubitus position. 3D-EAUS was performed using a 3D-EAUS system (Hawktype 2050, B-K Medical, Naerum, Denmark) with a rotating endoprobe housing two crystals covering 10–16 MHz (focal range 2–4.5 cm; diameter, 1.7 cm) and producing a 360˚ view and with an internal puller allowing longitudinal distances to be measured and a constructing a 3D image.

The fistula tract appeared as a hypoechoic tube-like lesion. If an external fistula opening was present, 2% hydrogen peroxide was introduced into the fistula track using a flexible intravenous cannula. The site of the IFO was identified as a sub-epithelial breach connected to an internal sphincter defect, or as a root-like budding which is in contact with, or is positioned inside the internal anal sphincter. This was according to the Cho criteria for identifying the IFO of an anal fistula tract [10]. The height of the IFO was measured starting from the anal verge. Preoperatively fistulae were classified as intersphincteric, low transsphincteric (involving the lower 1/3 of the sphincter complex), mid transsphincteric (involving the middle 2/3 of the sphincter complex), high transsphincteric (involving the highest 1/3 of the sphincter complex), suprasphincteric, or extrasphincteric. A fistula was considered complex if there were multiple fistula tracts or a mid/high transsphincteric, suprasphincteric, or extrasphincteric fistula tract. Low transsphincteric were classified as simple fistulas.

### Fecal incontinence (FI)

Preoperatively FI was assessed by the patient’s ability to hold solid stool, liquid stool, flatus, and soiling. This was determined by the patients’ history performed by a surgeon and was classified using the Parks FI classification [2]. Postoperative FI was evaluated by means of a paper questionnaire in December 2015 and by face-to-face contact or telephone in 2018.

### Surgical procedure

The patients arrived on the day of surgery. No bowel preparation or antibiotics were given prior to the surgery. The LIFT procedure was performed under general anesthesia. Essential steps of the procedure include incision at the intersphincteric groove, identification of the intersphincteric portion of the tract, thorough cleaning of the tract, ligation of intersphincteric tract close to the internal opening, removal of intersphincteric portion of the tract, core out of the external tract and the external opening, and suturing of the defect at the intersphincteric site of the external sphincter muscle, and the external opening was left open for discharge. The classic approach had to be modified if the tract could not be dissected safely (a very thick tract, too much fibrosis around the tract, proximal curving of the tract, and immediate branching of the tract at the intersphincteric site). The intersphincteric tract was then cut rather than dissected, and after removal of the intersphincteric portion of the tract, the tract was sutured. The suture was placed at the level of the internal sphincter muscle.

To verify that the fistula tract was closed, hydrogen peroxide was introduced into the IFO during the operation.

The patients were discharged the same day of surgery. Paracetamol and ibuprofen were prescribed for pain management.

### Follow-up

Patients were seen 2 weeks after the initial procedure by their surgeon.

The next consultations were at 4-weekly intervals until recurrence or complete healing had occurred.

The initial follow-up period with extensive questionnaires was from the first perianal fistula-related surgery up to December 2015.

The second follow-up was in September 2018 to evaluate recurrence and complaints only. The electronic patient files were reviewed and the checked for recurrences. If the patient was not seen during the previous 3 months, a telephone call was made.

A recurrence was defined as a persisting fistula opening after 3 months or a new fistula after the initial closure.

### Questionnaires

In December 2015, questionnaires regarding the current fistula-related perianal symptoms, complaints of fecal incontinence, and the impact of current fistula-related complaints on QOL (Procto-PROM) were sent out to all the participants. Current fistula-related complaints were defined as perianal pain, tenderness, or fistula-related pus secretion. If a patient did not return the first set questionnaires, they were sent a second set.

A patient-reported outcome measurement or PROM is a validated questionnaire used in a clinical trial or a clinical setting, where the responses are collected directly from the patient. Evidence shows that the systematic use of information from PROMs leads to better communication and decision-making between doctors and patients and improves patient satisfaction with care [11–13]. The validated Procto-PROM questions were divided in five categories: daily life, stool related, social life, coping, relationships, and intimacy. The maximum score per category is 10 points with a total score of 50 points. The higher the score, the more impact the symptoms have per category.

### Statistical analysis

Data were analyzed using the Statistical Packages for Social Sciences (SPSS, Chicago, IL, USA, version 22.0). Univariate analysis was performed on factors possibly associated with recurrence. Associations between groups were compared using the Chi-square, paired *t* test, or one-way ANOVA. A *p* value < 0.05 was considered to be statistically significant.

### Ethical issues

The study was approved by the Medical Ethical Committee of the VU University Medical Centre.

## Results

**Fig. 1:**
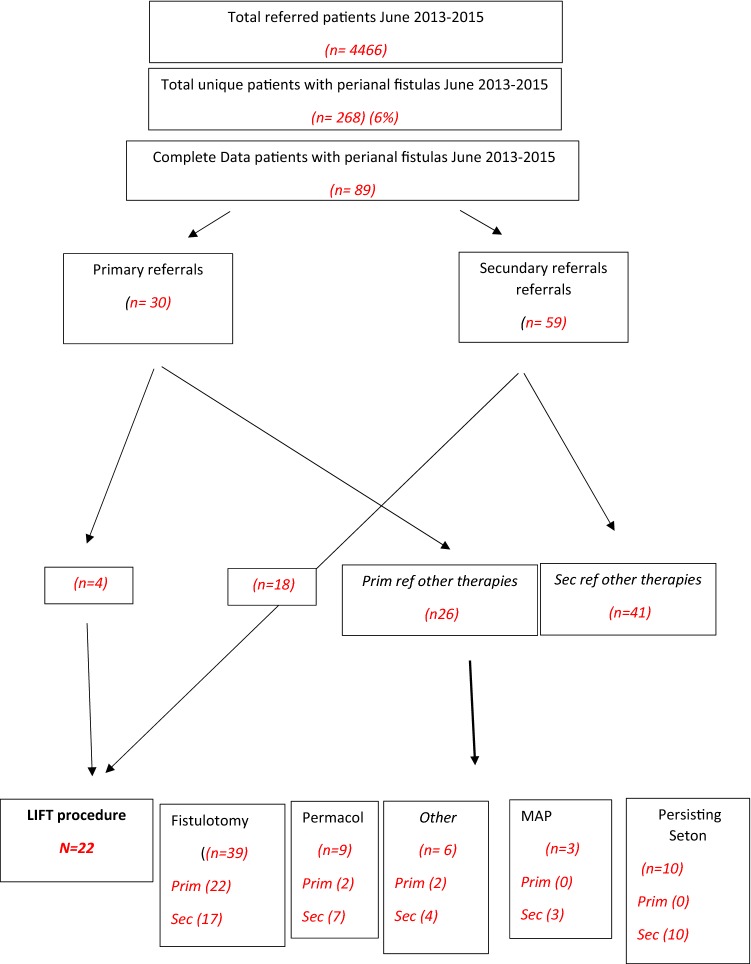
Flow diagram of treatment of patients with perianal fistulas in the period June 2013–Dec 2015

A total of 268 patients with perianal fistula were seen between June 2–13 and December 2015, from which 45 (17%) were treated with the LIFT procedure, 145(54%) with fistulotomy, 37 (14%) with a seton, 11 (4%) with fistulectomy, 11 (4%) with MAP, 11 (4%) with fistulectomy, 9 (3%) with Permacol, 7 (3%) with at excision of the external fistula opening, and 3 (1%) with bio-lift (Fig. 1).

All 45 patients were included (Table 1). There were 17 males (38%). Mean age was 40 years (range 24–67 years). Thirteen (29%) patients presented with a first fistula. Thirty-two patients (71%) had had previous fistula surgery, all had a seton and abscess drainage, and in 16 patients, 20 additional procedures were performed (MAP *n* = 6, fistulotomy *n* = 5, plug *n* = 4, fistulectomy *n* = 2, Permacol^®^ paste *n* = 1, excision external opening *n* = 1, and temporary stoma *n* = 1).

**Table 1:** Patients characteristics in 45 cryptoglandular fistulas

	All	No recurrence	Recurrence
All	45	18 (40%)	27 (60%)
Man	17	2 (11%)^ʈ^	15 (89%)^tt^
Woman	28	16 (57%)^t^	12 (43%)^t^
Age (mean, years)	40	39	40
History			
No previous fistula surgery	13 (29%)	5	8
Previous fistula surgery	32 (71%)	13	19
Seton + drainage	16	7	9
Seton + drainage + other^a^	16	6	10

^ʈ^*p* = 0.004

^a^Other: mucosal advancement flap 6, fistulotomy 5, plug 4, fistulectomy 2, Permacol^®^ paste 1, excision external opening 1, and temporary stoma 1

There were three diabetic patients using oral medication. Three patients smoked. One patient had a body mass index > 30 kg/m^2^. None had an enterostomy.

The mean operation time was 67 min (range 22–140 min). The first operations took longer due to the learning curve. There was no postoperative bleeding or infection.

The mean follow-up time for the questionnaires was 12 months [SD 0.5, (range 6–24 months)].

The classification of the fistulas is shown in Table 2. In 41 (91%) patients, a CF was present. The patients with mid and high transsphincteric fistulas all had secondary tracts or an extension higher than the internal opening.

**Table 2:** Classification and closure technique of the 45 fistulas related to recurrence

Type fistula	All	No recurrence	Recurrence
	45	18	27
Classification tracts			
1. Transsphincteric (low)	4 (9%)	1	3
2. Transsphincteric (mid)	5 (11%)	2	3
3. Transsphincteric (high)	34 (76%)	14	20
4. Ano-introital	2 (4%)	1	1
Simple or complex			
Simple (1)	4 (9%)	1	3
Complex (2–4)	41 (91%)	17	24
Location IFO			
Anterior	27 (60%)	12	15
Posterior	14 (31%)	4	10
Right lateral	2 (4%)	1	1
Left lateral	2 (4%)	1	1
Height IFO	(16.1 mm)	(13.3 mm)	(18.5 mm)
≥ 20 mm	13	3 (23%)^#^	10 (77%)^#^
≥ 15 mm	28	8 (29%)^Δ^	20 (71%)^Δ^
Closure technique			
Suture	16 (35%)	8	10
Ligation	29 (65%)	11	18

*IFO* internal fistula opening

^#^*p* < 0.03

^Δ^p < 0.03

### Results

#### Questionnaires evaluation: December 2015

From the 45 patients included, 32 (71%) patients returned the questionnaire. Three patients refused to fill in the questionnaire and ten patients could not be contacted. The mean follow-up at this point was 12 months (SD 0.5, median 13 months, range 7–24 months)

##### FI

Preoperatively, eight patients had existing FI, seven had a history of fistula-related surgery, and one had a previous seton and abscess drainage only. Three women and one man were incontinent for solid stool (Parks 4), two men and one woman for liquid stool (Parks 3), and one man for flatus (Parks 2). Three men had soiling due to a keyhole deformation. Postoperatively, their Parks score was unaltered and no additional symptoms of soiling were reported.

##### QOL (Prom)

There was a negative effect of a CF (simple 5.8 and complex 13.9 (*p* < 0.01) and a trend with recurrence of the fistula (no recurrence 8.8 and recurrence 14.9 *p* = 0.07) on the QOL.

### Recurrences

**Fig. 2:**
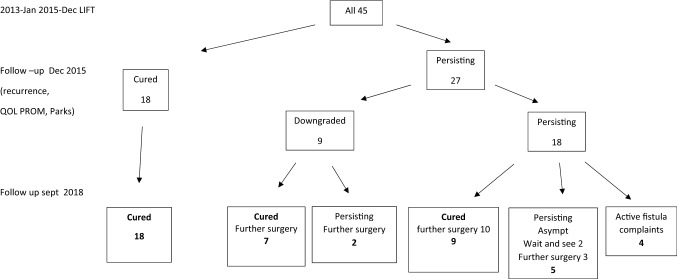
Flow sheet of Patients and follow-up of the questionnaires in 2015 and telephone call long-term follow-up in 2018. The initial cure after LIFT was 18 (40%) in 2015. In 2018, further treatment has led to cure in 34 (75%), asymptomatic fistulas in 7 (16%), and persisting active fistulas in 4 (9%)

Figure 2 shows a flow sheet of the results. Recurrences occurred after a mean of 3 months (0.5–12 months).

#### Evaluation: December 2015

Of the 45 patients, 18 (40%) were successfully cured after the first LIFT procedure and 27 (60%) experienced a recurrence. The demography and fistula classification of the patients with and without recurrence are shown in Tables 1 and 2. There were no statistically significant differences between the two groups concerning, age, previous fistula treatment, the complexity of fistula tract(s), the surgical technique of suture versus ligation, the clockwise orientation of the IFO, and the duration of the placement of a seton.

Men more had more recurrences then women (89% and 43%, *p* < 0.04).

A higher IFO had a higher chance of recurrence (13.3 mm in non-recurrence and 18.5 mm in recurrence, *p* = 0.05), especially when the IFO was situated ≥ 15 mm above the ano-dermal junction (RR = 0.34% CI 0.12–0.99 *p* = 0.030).

The mean height of the IFO in men was 17.3 mm and women 15.1 mm (*p* = 0.09).

Of the 27 recurrences, 9 (33%) were downgraded (on clinical examination and EUS) to a lower transsphincteric (1) or intersphincteric (8) fistula without an extra branch (Table 3).

**Table 3:** Recurrences and follow-up in 27 patients with cryptoglandular fistulas

Pre-LIFT fistula classification in patients with recurrence		Post-LIFT fistula classification in patients with recurrence Dec 2015		Post-LIFT Sept 2018			
Fistula type	All 27	Unaltered 18 (67%)	Downgraded 9 (33%)	Treatment follow-up	Cured 17 (61%)	Asym 8 (29%)	Fistula 3 (10%)
Transsphincteric (low)	3	2	*1 inter*	1 S, F	1		
				1 S, re-lift, PP, S		1	
				*1 MAP, asympt.*		1	
Transsphincteric (mid)	3	1	*2 inter*	1 bio-lift	1	1	
				*1 F*	1		
				*1 asymp.*			
Transsphincteric (high)	20	14	*5 inter*	14^a^ (4^b^)	6 (3^b^)	4 (1^b^)	4
			*1 trans mid*	*2 S (2x), F,*	2		
				*1 S, PP, asymp.*		1	
				*1 F*	1		
				*1 PP*	1		
				*1 F*	1		
Ano-introital	1	1	0	Re-lift, bio-lift	1		

The 9 downgraded fistulas: 7 (78%) cured and 2 (22%) asymptomatic; 18 unaltered fistulas 9 (50%) were cured, 5 (28%) asymptomatic, and 4 (22%) persistent fistula

*S* seton, *F* fistulotomy, *MAP* mucosal advancement, *PP* Permacol paste

^a^Any or combination of techniques: seton, 2 re-LIFT, 2 bio-LIFT, PRP, PP, and F (1 no surgery, 4 patients one, 4 patients two, and 4 patients three surgeries)

^b^Patients with (Bio)Lift

The *italic* fistulas are the downgraded fistulas

#### Long-term evaluation: September 2018

All but two patients could be contacted (96%). Of the patients who were lost to follow-up, one had a persisting asymptomatic fistula and one had an active fistula without complaints who moved abroad. Their last follow-up was in 2017.

The mean follow-up was 45 months (median 46 months; range 40–57 months).

Both the cure rate and the amount of subsequent procedures did not differ between downgraded or unaltered fistulas (Table 3, Fig. 2).

After the initial cure after LIFT of 18 (40%) patients in 2015, further surgery with the other techniques lead to cure in 34 (75%), and asymptomatic fistulas in 7 (16%) and 4 (9%) in 2018. Two patients treated additionally with a fistulotomy developed minor soiling.

## Discussion

The success rate for the initial cure in our study was low: 40%. Of the 27 recurrences, downgrading occurred in 9 (33%). All recurrences were subsequently treated with (several) subsequent procedures (Table 3, Fig. 2), which resulted in a healed fistula in another 16 patients and asymptomatic fistulas in 7 patients. Looking at the original group of 45 patients, ultimately 75% were cured, 16% had asymptomatic fistulas, and 9% had persisting fistulas.

Although, in 60% of the cases, the fistula did not resolve after the first operation, down-staging of the complexity of the fistula was obtained in 30%. The importance of downgrading is that the remaining fistula tract is easier to treat. In general, a fistulotomy can be performed with little risk of FI.

Other centers have had the same experience [14]. There was a trend towards a higher healing rate and less subsequent procedures between the downgraded and unaltered fistulas.

Our results seem disappointing compared to the results in the literature, where the success rates are generally higher. Table 4 shows a review of the published studies including > 35 patients [14–25]. A systematic review in 2016 [26] evaluated articles with perineal procedures and found 19 appropriate articles with an overall success rate of 51–94%. Ten of these studies had less than 35 patients, five were prospective, and only three studies had a follow-up longer than 1 year.

**Table 4:** LIFT procedure in series > 35 patients

Author	Year	*N* (%male)	Complex Fistula (%)	Other fistula classification complex	Previous surgery (%)	Previous seton (%)	Success rate (%)	Follow-up (months)
Bleier	2010	39 (51%)	25	hs, high	74	–	57	5
Shanwani^c^	2010	45 (71%)	27	>30% Sphincter	11	–	64 r	9 (2–16)
Tan	2011	93 (83%)	58	High and mt	28	–	78	5.8
Abcarian	2012	40 (?)	–	–	75	–	74	4.2
Wallin	2012	93 (61%)	17 hs 26 mt	hs/mt >35% sphincter	32	92	40 in 57^a^	19 (4–55)
Liu	2013	38 (74%)	–	Length of fistula tract	18	76	62 in	26 (3–44) 68% > 12
Bastawrous	2015	56 (76%)	–	–	52	55	71 in 65 r	4.8
Parthasarathi^c^	2015	167 (81%	All	>1/3 Sphincter or branches	33	–	94	12 (4–22)
Schultze^c^	2015	75 (68%)	All	>30% Sphincter	48	–	88 r	14.6
Chen	2017	43 (74%)	33	hs/mt	28	–	84^a^	26 (13–63)
Xu	2017	55 (64%)	All	hs/mt	100	–	60 r	16
Wen^b^	2018	62 (69%)	–	41 ht, 4 H int.sph, 17 Ant Fe	29	–	84	24 (12–51
Sun	2019	70 (84%)	All	High: above subcut ext sphincter	24	–	81 67 r	16 (4.5–68)
Mijnsbrugge	2019	45 (38%)	91	>1/3 Sphincter or branches height of IO	71	71	40 75^a^	45 (40–57)

– not clearly indicate, *hs* horseshoe, *ht* high transsphincteric, *mt* multiple tracts, *io* internal opening, *in* initial cure, *r* cure after recurrence

^a^Cure or downgraded with subsequent surgery

^b^Modified LIFT

^c^Prospective

What is the explanation for such a diversity in success?

First, the definition of complex and simple fistulas and the number of included CF. This is not always mentioned. The definition involves the height of the fistula and side branches. We had 91% CF; almost all transsphincteric fistulas were very high (involving the highest 1/3 of the sphincter complex) (Table 2). Furthermore, most investigators use the lowest 1/3 of the sphincter complex as a starting point for high fistulas. Finally, our fistulas had extensions or side branches above the IFO. Recurrences have been related to complexity of fistulas [24, 27, 28].

Second, the mean follow-up in the literature did not always reach 6 months and even less studies had a follow-up of more than 1 year. Although most recurrences occur within 6 months, many individual patients were followed for less than 6 months.

Third, the definition of success differs between different studies. The initial closure is often noted as success and not the corrected number after subsequent recurrences. Furthermore, whether cure occurred after subsequent treatments is not always clear.

Fourth, we had a high percentage of previously operated patients many of whom had undergone more than 1 procedure. Recurrence has been related to past fistula surgery [24, 27, 28].

Besides the previous surgery, several other factors have been associated with recurrence. Unexplored secondary tracts are a well-known cause [27]. With EUS or magnetic resonance imaging (MRI) before surgery, missed tracts are generally avoided. There is some discussion about surgery being the golden standard to evaluate secondary tracts, but studies with anal ultrasound and MRI have demonstrated that tracts found with these modalities can be missed during surgery. EAUS is an easy to use bedside tool providing excellent visualization of the fistula tracts [29]. The length of the fistula [15], lateral localization of the IFO [28], diabetes, smoking, and obesity [15, 18, 22, 27] have also been mentioned as possible causes of recurrence or failure.

The previous treatment with a seton is still a matter of debate. Epithelialization of the tract as a result of seton placement seems logical, but has not been demonstrated [30] and has been associated with failure [21].

Antibiotics were not used in our study. The role of concomitant infection is controversial. The use of antibiotics or core out [22, 24, 27] has been successful in some cases, but is not generally applied. Most studies do not mention the use of antibiotics and no clear evidence exists concerning recurrence. [24].

The only predictor which we found for recurrence was the height of the IFO. We believe that the height of the IFO is crucial in the definition of the complexity of the fistula. In our study, 76% of the fistulas were classified as high transsphincteric. The unexpected finding that men had more recurrences than women was possibly due to the higher IFO in men. Although persistence or recurrence is disappointing, subsequent surgery can often cure the fistula [14, 15].

The LIFT procedure did not lead to genuine FI; two patients developed soiling due to subsequent fistulotomy after downgrading with LIFT. Other studies report similar experiences [19, 21, 23].

The PROM (QOL) score was lower in patients with recurrence and a CF. We used a PROM questionnaire, since this is now a routine procedure in our clinic before and after treatment. The FI QOL scale is also a good tool [31], but does not reflect the real disease burden with problems in fistula surgery.

This retrospective study has some limitations. Although our prospective database is very extended and precise, the questionnaires were not applied before surgery at that time. Furthermore, the response of 71% to the questionnaires is suboptimal. However, in 2018, all records were studied again and checked for recurrences, FI and soiling; when patients had not been seen for than 3 months, they were reached by telephone

The importance of this study is that it shows again that initial success rates of treatment in patients with perianal fistulas are overrated and studies should be repeated by the other groups to confirm the success rate. The commonly heard saying that complicated fistulas have a recurrence of 50% independently of the technique used seems true. Furthermore, this study shows that, by combining techniques, cure or asymptomatic fistulas can be achieved in almost all patients.

## Conclusions

The only predictive factor for recurrence with LIFT was the height of the IFO especially if located ≥ 15 mm from the anal verge.

Continence was preserved and incontinence did not worsen in patients who had already complaints. Recurrences and complexity of the fistula were negatively associated with FI and QOL.
